# Changes in the quality of flaxseed oil powder produced by incorporating with microcrystalline cellulose and thyme

**DOI:** 10.1016/j.heliyon.2023.e18562

**Published:** 2023-07-22

**Authors:** Mahsa Farhoudpour, Sodeif Azadmard-damirchi, Mehdi Gharekhani, Narmela Asefi

**Affiliations:** a Department of Food Science and Technology, Tabriz Branch, Islamic Azad University, Tabriz, Iran; b Department of Food Science and Technology, University of Tabriz, Tabriz, Iran

**Keywords:** Oxidation, Essential fatty acid, Stability, Quality

## Abstract

Flaxseed oil is a high nutrition oil, rich in ω-3 fatty acid, tocopherols and phenolic compounds. However, it is prone to oxidation due to its high unsaturation which needs pretreatments to be easily introduce to the market. In this study, flaxseed oil was converted to powder form by mixing with microcrystalline cellulose (MC) and thyme powder to produce flaxseed oil powder. For this purpose, 3 different levels of thyme powder (5%, 10% and 15% of oil) were mixed with oil, followed by mixing with different proportions of MC (50:50, 50:75, 50:100 (oil:MC)) and stored for 90 days at 25 °C and 4 °C. Results indicated that the total phenolic compounds (23.2–91.2 mg GAE/100 g), chlorophyll (9–63.6 mg/kg), and carotenoid (4.4–9.9 mg/kg) contents increased with the incorporation of thyme powder into the flaxseed oil on the first day in 25 °C and 4 °C. Also during 90 days storage, phenolic compound (21.8%), chlorophyll (32.5%) and carotenoid (24%) decreased in both 25 °C and 4 °C temperatures. The results confirmed that adding thyme powder to samples decreased acidity and peroxide value in compare with control. Using thyme as a natural antioxidant and also transforming the oil to powder form by MC increased the oxidative stability in compare with control. The produced high stable flaxseed oil powder has the potential to be used directly on food products like salads or to be used in different food formulations to fortify them with natural antioxidants and ω-3 essential fatty acids.

## 1 Introduction

Flaxseed (*Linum usitatissimum* L.), which is also known as linseed, is an annual crop that has an oval and flat seed in golden yellow to dark brown colors [1]. These seeds contain relatively high amount of oil (50–60%), rich in α-linolenic acid, an ω-3 essential fatty acid. The flaxseed oil (FO) also contains phenolic compounds with antioxidative properties (lignans, ferulic acid, and ρ-cumaric acid), tocopherols, and carotenoids, all of which make FO a functional food with health-promoting effects [[2], [3]]. FO has high amount of unsaturated fatty acids, which makes it very susceptible to oxidation; this oxidation results undesirable changes in taste and odor, and also a decrease in nutritional value [4]. Oxidation can be retarded using natural or synthetic antioxidants. However, natural antioxidants are more popular due to the health concern of synthetic antioxidant applications [5].

In recent years, herbs have been used as natural antioxidants and also as flavor components in vegetable oils [6]. *Thymus vulgaris* L., which is also known as common thyme, is an aromatic plant with health-promoting properties, such as anti-inflammatory, anti-proliferative, and also anti-microbial effects. It contains high amounts of phenolic and bioactive compounds such as rosmarinic acid, luteolin, caffeic acid, apigenin, and their derivatives, which give it free radical scavenging activity and antioxidant properties [7]. Considering its antioxidative property, thyme can be used as a natural antioxidant to prevent oil oxidation [8].

Another alternative method to retard oil oxidation is microencapsulation, which creates a physical barrier surrounding that prevents oxygen contact with oil [9]. Spray drying, complex coacervation, and extrusion are popular methods for microencapsulating of oil containing ω-3 fatty acids [10]. However, these methods can be energy-consuming and costly. A simple method to produce powdered oil is mixing oil with absorbent powders such as maltodextrin and microcrystalline cellulose (MC). MC is made from materials composed of cellulose, such as wood and cotton. It is conventionally produced from acid hydrolysis and partial depolymerization of pure cellulose. It can be used as an emulsifier, stabilizer, and viscosity enhancing agent in the food industry [11].

Due to the low oxidative stability and high nutritional value of FO, this study aimed to produce and evaluate the quality of flaxseed oil powder by mixing and incorporating it with MC and enhancing oxidative stability by the incorporation of thyme leaf powder as a natural antioxidant.

## 2 Materials and methods

### 2.1 Materials

Flaxseeds and thyme powder were purchased from the local market (Tabriz, Iran). Microcrystalline cellulose was supplied from Hirania industries (India). Other chemicals used in this study were obtained from Sigma-Aldrich (Steinheim, Germany).

### 2.2 Methods

#### 2.2.1 Flaxseed oil extraction

Flaxseed oil was extracted from the cleaned flaxseeds using a screw press (Model 85 mm, 10 MPa) below 40 °C [12]. The extracted oil was kept in a dark booth in the refrigerator (−18 °C) until use.

#### 2.2.2 Flaxseed oil powder preparation

Flaxseed oil samples were mixed thoroughly with thyme powder at the levels of 5%, 10%, and 15% of oil. Then microcrystalline cellulose (MC) at the levels of 50:50, 50:75, and 50:100 (oil: MC) were added to each mixture, and prepared nine samples (Table 1). The prepared samples were filled into PET cups and divided into two groups, one group stored at room temperature (25 °C) and the other group stored at the refrigerator (4 °C) both in dark condition. Flaxseed oil (control A) and also flaxseed oil mixed with MC with a ratio of 50:75, without adding thymus (control B), were used as control samples.

**Table 1:** Composition of prepared samples.

sample	composition	sample	composition
T1	50gr oi + 50gr MC + 2.5gr thyme	T6	50gr oil + 75gr MC + 7.5gr thyme
T2	50gr oil + 50gr MC + 5gr thyme	T7	50gr oil + 100gr MC + 2.5gr thyme
T3	50gr oil + 50gr MC + 7.5gr thyme	T8	50gr oil + 100gr MC + 5gr thyme
T4	50gr oil + 75gr MC + 2.5gr thyme	T9	50gr oil + 100gr MC + 7.5gr thyme
T5	50gr oil + 75gr MC + 5gr thyme	Control A	Flaxseed oil
Control B	Flaxseed oil +75gr MC		

MC: microcrystalline cellulose.

#### 2.2.3 Oil extraction from the samples

For the analysis of oil quality during storage, oil was extracted from the flaxseed oil powder samples using petroleum benzene as a solvent. Then the solvent was removed using a rotary evaporator at temperature below 40 °C, According to the method described by Savage et al. [13].

#### 2.2.4 Determination of acid value

The acid value of samples was measured according to the American Oil Chemists' Society official method on days 1, 30, 60, and 90 [14]. Added 50 ml ethanol on 1 g of sample and then titrated using 0.1 N Sodium hydroxide. Acidity value calculated as equation (1):

(1) $$AV=\left(\frac{N\times V}{W}\right)\times 28.2$$

where N is normality of Sodium hydroxide, V is volume of Sodium hydroxide, W is the sample weight (g).

#### 2.2.5 Determination of peroxide value

The peroxide value (PV) was determined every 30 days (days 1, 30, 60, and 90) according to the American Oil Chemists' Society official method [14]. Adding 30 ml of 3:2 acetic acid/chloroform (v/v) solution and 0.5 ml of saturated potassium iodide (KI) on 1 g of extracted oil. After shaking for 1 min, 30 ml of water added to the mixture. 0.5 ml aliquot of 1% (w/v) starch indicator was then added. The resulting solution was titrated using 0.001 N sodium thiosulfate until the purple color disappeared. Peroxide value calculated as equation (2):

(2) PV=(S−B)×N×1000/W

where S is volume of sodium thiosulfate, B is the volume of sodium thiosulfate of the blank, N is the normality of sodium thiosulfate solution and W is the sample weight (g).

#### 2.2.6 Determination of total phenolic compounds

The total phenolic compounds were determined every 30 days (days 1, 30, 60, 90) using the method described by Gouvinhas et al. [15]. Total phenolic compounds determined using the Folin-Ciocalteu (FC) reagent. 0.1 ml of sample was dissolved in 1 ml of n-hexane, followed by addition of 3 ml of sodium carbonate (10%). Then, 0.75 ml of previously diluted (10 fold) FC reagent was added and the mixture was kept at room temperature for 90 min. The absorbance of the mixture was measured at 710 nm using Ultraviolet–Visible spectrophotometer.

#### 2.2.7 Determination of chlorophyll and carotenoid contents

Carotenoids and chlorophyll contents were measured by Spectrophotometer (UNICO, model 2100, USA) on days 1, 30, 60, and 90 using the method of Minguez-Mosquera et al. [16]. Samples were completely dissolved in 20 mL cyclohexane. The absorbance of the solution was determined at wavelengths of 470 and 670 nm for carotenoids and chlorophylls, respectively. Calculation of the pigment content is by equations (3), (4)):

(3) $$Carotenoids\;\left(mg/kg\;of\;oil\right)=\left(A470\times 10^{6}\right)/\left(2000\times 100\times d\right)$$

(4) $$Chlorophyll\;\left(mg/kg\;of\;oil\right)=\left(A670\times 10^{6}\right)/\left(613\times 100\times d\right)$$

where A is the sample’s absorbance, 2000 is the carotenoids extinction coefficient, 613 is the chlorophyll extinction coefficient, and d is the thickness of the spectrophotometer cell (1 cm).

#### 2.2.8 Determination of fatty acid composition

The fatty acid content of samples was determined by gas chromatography (GC). Before GC analysis, methyl esterification was used as a pre-treatment of the oils. The fatty acid methyl ester was analyzed using a GC (Agilent 8790B) coupled with an FID detector. Temperature was upper than 100 °C and the instrument and injection parameters were set according to the method defined by Fathi-Achachlouei et al. [17]**.**

#### 2.2.9 Statistical analysis

The results of experiments were expressed as a mean value of three replication ± standard deviation. A completely randomized design was performed using SPSS 16.0 (SPSS Inc., Chicago, IL) for the analyze of data. Duncan’s test at a 5% significance level was used to test the significance of differences.

## 3 Results and discussion

### 3.1 Acid value

**Fig. 1:**
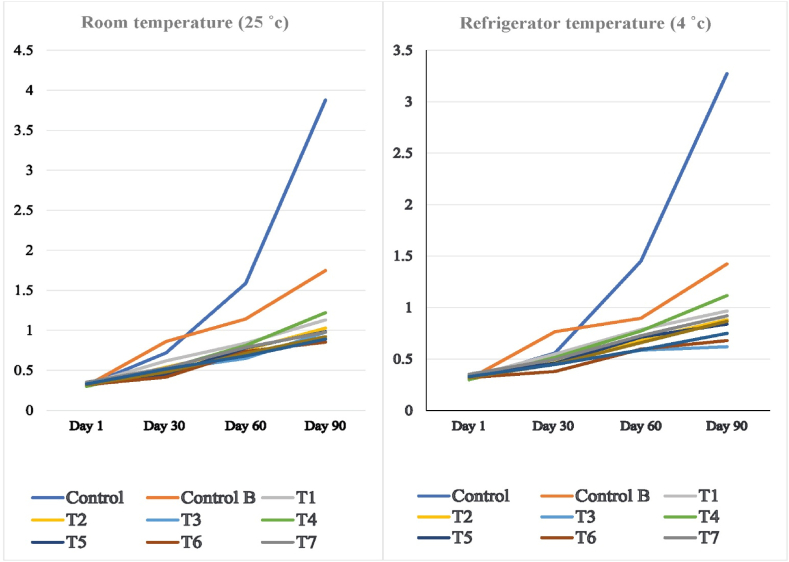
Changes in the acidity (mg KOH/g) of flaxseed oil powder during storage. Samples: T1 (50gr oil + 50gr microcrystalline + 2.5gr thyme), T2 (50gr oil + 50gr microcrystalline + 5gr thyme), T3 (50gr oil + 50gr microcrystalline + 7.5gr thyme), T4 (50gr oil + 75gr microcrystalline + 2.5gr thyme), T5 (50gr oil + 75gr microcrystalline + 5gr thyme), T6 (50gr oil + 75gr microcrystalline + 7.5gr thyme), T7 (50gr oil + 100gr microcrystalline + 2.5gr thyme), T8 (50gr oil + 100gr microcrystalline + 5gr thyme), T9 (50gr oil + 100gr microcrystalline + 7.5gr thyme), control A (Flaxseed oil) and control B (flaxseed oil + microcrystalline with the ratio of 50:75).

Triacylglycerol hydrolysis can happen for different reasons, such as heat, stress, or enzyme activity in the presence of moisture, which cause the formation of free fatty acids (FFA) [18]. The results of high content of FFA are several severe problems in oil and food containing oil, such as low smoke point, poor oxidative stability, or changes in flavor [19]. Generally, FFA is determined by acidity, and high acidity is unfavorable in vegetable oils. There are different ways to control acidity, such as storage at lower temperatures and also transforming oils to powder form [20]. In the present study, bulk oil (control A) had the highest acidity, and transforming the oil to powder form had a protective effect against the FFA increase. The acidity of all samples increased during storage. However, the rate of acidity increase was slower in samples stored in the refrigerator (Fig. 1). At the end of storage, the acidity of bulk oil (control A) was 2.3 times higher than the oil powder sample only with MC (control B) which shows the inhibition effect of microcrystalline cellulose on the hydrolysis of triacylglycerol and formation of FFA. Similar results have been reported for the inhibition effect of microencapsulation of flaxseed oil with chitosan or sodium alginate on acidity increase. It has been reported that encapsulation with chitosan was more effective in acidity control in flaxseed oil than with sodium alginate [21].

Although the incorporation of thyme powder in to the oil powder samples slowed down the FFA increase rate (Fig. 1). Oil powder samples incorporated with thyme powder were not superior to each other (*p* > 0.05), and all prevented an increase in acidity to some extent. It has been reported that the incorporation of thyme essential oil into vegetable oils such as sunflower, corn, and grape seed oils decreased the acidity after accelerated storage of samples (4 days at 60 °C) [22]. Thyme powder may absorb the moisture present in the medium, and also, their bioactive compounds, such as essential oils, can prevent enzymatic hydrolysis, and therefore, the formation of FFA can be slower.

### 3.2 Peroxide value

**Fig. 2:**
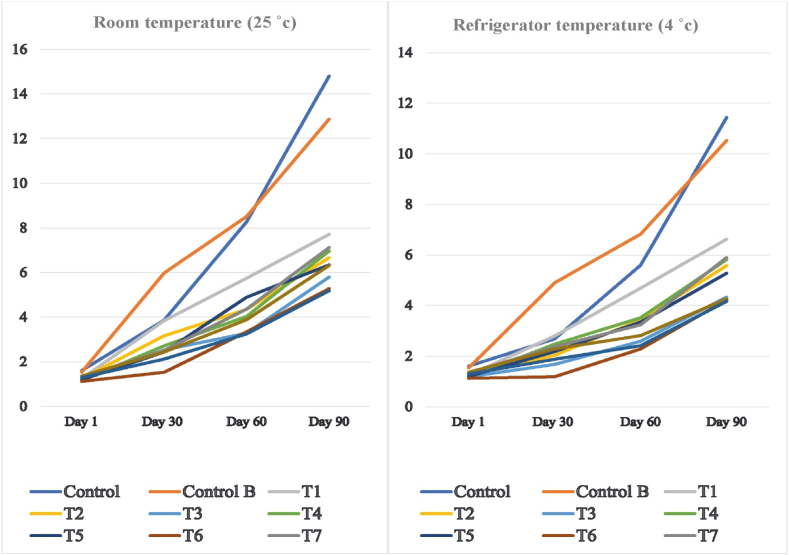
Changes in the peroxide (meq $O_{2}$/kg) of flaxseed oil powder during storage. Samples: T1 (50gr oil + 50gr microcrystalline + 2.5gr thyme), T2 (50gr oil + 50gr microcrystalline + 5gr thyme), T3 (50gr oil + 50gr microcrystalline + 7.5gr thyme), T4 (50gr oil + 75gr microcrystalline + 2.5gr thyme), T5 (50gr oil + 75gr microcrystalline + 5gr thyme), T6 (50gr oil + 75gr microcrystalline + 7.5gr thyme), T7 (50gr oil + 100gr microcrystalline + 2.5gr thyme), T8 (50gr oil + 100gr microcrystalline + 5gr thyme), T9 (50gr oil + 100gr microcrystalline + 7.5gr thyme), control A (Flaxseed oil) and control B (flaxseed oil + microcrystalline with the ratio of 50:75).

Flaxseed oil is highly susceptible to oxidation due to its highly linolenic (18:3) and linoleic (18:2) fatty acids content [23]. The oxidation can be promoted by peroxidant such as Fe and Cu, high temperature, moisture, oxygen, and light exposure. The application of antioxidants in vegetable oils is a primary way to control oxidation. Also, storage at low temperatures can delay and prevent oxidation. Results showed that the rate of peroxide value (PV) increment in samples stored in the refrigerator was lower than those stored at room temperature (Fig. 2). Mixing with microcrystalline cellulose not only had a detrimental effect on the oxidative stability of flaxseed oil, but also increased its stability even during storage. Bulk oil (control A) had the highest PV among the oil samples. This could be attributed to reduced oxygen contact with oil by the physical barrier of microcrystalline cellulose. With an increase in the microcrystalline cellulose content, the protecting effect against oxidation was increased. Also, thyme powder reduced the progress of PV in the flaxseed oil powder. Similar results were reported on retarded oxidation of spray-dried flaxseed oil emulsions with whey protein and also encapsulated flaxseed oil with gelatin-gum Arabic compared to the bulk oil [24,25]. It has been reported that the freeze-dried microcapsules of flaxseed oil with chickpea or lentil protein isolates mixed with maltodextrin were more stable against oxidation compared to the bulk oil [26].

With an increase in thyme powder content, the PV of the samples decreased. Thyme powder, as a source of natural antioxidants, could retard the formation of oxidation products in vegetable oils. Thyme powder addition increased the phenolic compounds and carotenoids in the sample, which have antioxidative properties. Similar results have been reported for enhanced oxidative stability by the incorporation of thyme extract into vegetable oils, such as soybean [27,8]. Samples containing the highest thyme content (15% of the oil content) meet the acceptable peroxide criteria for ω-3 rich products At the end of 90 days of storage (≤5 meq/kg oil) [28].

### 3.3 Total phenolic content

Phenolic compounds are one of the vital antioxidation compounds for maintaining the oil quality during storage. Phenolic acids such as caffeic, ρ-cumaric, and ferulic acids comprise the main fraction of phenolic compounds in flaxseed oil [29,30]. Results indicated that the total phenolic content of samples decreased during the storage (Table 2). This could be due to the degradation and oxidation of the phenolic compounds caused by hydrolytic enzyme activity, oxygen, and temperature during the storage, which occurred more slowly at a lower temperature (4 °C). The total phenolic compounds of flaxseed oil (control A) was 23.92 (mg GAE/100 g), which decreased up to 50% (reached to12.35 mg GAE/100 g) during storage for 90 days at the refrigerator.

**Table 2:** Changes in the phenolic compound (mg GAE/100 g) of flaxseed oil powder during storage.

Sample	Room temperature (25 °C)				Refrigerator temperature (4 °C)			
	Day 1	Day 30	Day 60	Day 90	Day 1	Day 30	Day 60	Day 90
T1	^Ha^ 34.1 ± 0.03	^Hb^ 29.4 ± 0.08	^Hc^ 21.8 ± 0.04	^Hd^ 18.4 ± 0.02	^Na^ 34.1 ± 0.08	^Hb^ 32.1 ± 0.04	^Ec^ 27.5 ± 0.05	^Gd^ 22.1 ± 0.07
T2	^Ea^ 57.2 ± 0.03	^Db^ 55.2 ± 0.03	^Cc^ 48.7 ± 0.02	^Dd^ 45.7 ± 0.05	^Ea^ 57.2 ± 0.02	^Db^ 56.4 ± 0.08	^Cc^ 52.3 ± 0.07	^Dd^ 48.3 ± 0.04
T3	^Ba^ 89.6 ± 0.06	^Bb^ 82.9 ± 0.04	^Bc^ 74.2 ± 0.05	^Bd^ 65.3 ± 0.03	^Ba^ 89.6 ± 0.04	^Bb^ 86.6 ± 0.02	^Bc^ 75.3 ± 0.02	^Bd^ 69.2 ± 0.05
T4	^Ga^ 35.1 ± 0.07	^Ib^ 28.4 ± 0.05	^Gc^ 22.8 ± 0.05	^Gd^ 18.9 ± 0.10	^Ha^ 35.1 ± 0.08	^Ib^ 30.1 ± 0.04	^Ec^ 25.6 ± 0.06	^Hd^ 21.3 ± 0.10
T5	^Fa^ 55.4 ± 0.04	^Eb^ 52.6 ± 0.06	^Dc^ 47.2 ± 0.03	^Ed^ 43.5 ± 0.05	^Ga^ 55.4 ± 0.04	^Fab^ 53.3 ± 0.04	^Dc^ 46.3 ± 5.15	^Ebc^ 46.8 ± 0.07
T6	^Aa^ 91.2 ± 0.06	^Ab^ 88.2 ± 0.05	^Ac^ 83.2 ± 0.02	^Ad^ 71.3 ± 0.04	^Aa^ 91.2 ± 0.05	^Ab^ 88.4 ± 0.03	^Ac^ 82.08 ± 0.02	^Ad^ 71.9 ± 0.05
T7	^Ga^ 35.1 ± 0.05	^Gb^ 31.7 ± 0.07	^Fc^ 23.1 ± 0.03	^Gd^ 18.8 ± 0.05	^Ga^ 35.1 ± 0.07	^Gb^ 32.5 ± 0.05	^Ec^ 25.7 ± 0.05	^Id^ 20.3 ± 0.03
T8	^Da^ 58.2 ± 0.08	^Fb^ 51.6 ± 0.05	^Ec^ 44.5 ± 0.03	^Fd^ 40.2 ± 0.04	^Da^ 58.2 ± 0.05	^Eb^ 53.7 ± 0.05	^CDc^ 49.5 ± 0.08	^Fd^ 45.7 ± 0.05
T9	^Ca^ 88.4 ± 0.02	^Cb^ 80.5 ± 0.05	^Bc^ 74.3 ± 0.07	^Cd^ 65.09 ± 0.03	^Ca^ 88.4 ± 0.03	^Cb^ 82.1 ± 0.06	^Bc^ 74.8 ± 0.05	^Cd^ 66.2 ± 0.07
Control A	^Ia^ 29.3 ± 0.03	^Jb^ 22.8 ± 0.02	^Jc^ 16.6 ± 0.05	^Id^ 10.5 ± 0.08	^Ia^ 23.9 ± 0.05	^Jb^ 23.1 ± 0.02	^Fc^ 17.4 ± 0.06	^Kd^ 12.3 ± 0.04
Control B	23.2 ± 0.05 ^Ja^	^Kb^ 20.06 ± 0.06	^Ic^ 16.9 ± 0.06	^Jd^ 9.2 ± 0.04	^Ja^ 23.2 ± 0.06	^Kb^ 20.6 ± 0.05	^Fc^ 18.4 ± 0.04	^Jd^ 12.8 ± 0.05

Samples: T1 (50gr oil + 50gr microcrystalline + 2.5gr thyme), T2 (50gr oil + 50gr microcrystalline + 5gr thyme), T3 (50gr oil + 50gr microcrystalline + 7.5gr thyme), T4 (50gr oil + 75gr microcrystalline + 2.5gr thyme), T5 (50gr oil + 75gr microcrystalline + 5gr thyme), T6 (50gr oil + 75gr microcrystalline + 7.5gr thyme), T7 (50gr oil + 100gr microcrystalline + 2.5gr thyme), T8 (50gr oil + 100gr microcrystalline + 5gr thyme), T9 (50gr oil + 100gr microcrystalline + 7.5gr thyme), control A (Flaxseed oil) and control B (flaxseed oil + microcrystalline with the ratio of 50:75).

The incorporation of thyme powder into formulations increased the total phenolic compounds of samples. With increasing the thyme content, the total phenolic compounds of samples increased significantly (*p* < 0.05). The incorporation of 15% thyme powder into the flaxseed oil increased the phenolic compounds by 3.75 times (88.48–91.23 mg/kg oil). Thyme leaf contains high content of natural antioxidants, such as phenolic compounds. Ferulic acid, quercetin, gallic acid, ρ-coumaric acid, pyrogallol, ellagic acid, ρ-hydroxy benzoic acid, and vanillin are the main phenolics in thyme extract [29,30].

### 3.4 Chlorophyll and carotenoid contents

Chlorophylls and carotenoids play an essential role in the oxidative stability of oils. Chlorophylls prevent auto-oxidation in the dark condition, while promoting photo-oxidation in the presence of light. Carotenoids act as antioxidants and avoid photo-oxidation by filtering the light and inactivation and quenching of sensitizers [31]. The pigment content of all samples decreased significantly (*p* < 0.05) during the storage (Table 3, Table 4). The chlorophyll and carotenoid contents of the flaxseed oil were about 9 and 4.5 mg/kg, respectively. The chlorophyll and carotenoid contents decreased by about 23% and 48% at the end of 90 days of storage at 4 °C, respectively, which could be due to the decomposition and oxidation of these compounds.

**Table 3:** Changes in the chlorophyll (mg/kg) of flaxseed oil powder during storage.

Sample	Room temperature (25 °C)				Refrigerator temperature (4 °C)			
	Day 1	Day 30	Day 60	Day 90	Day 1	Day 30	Day 60	Day 90
T1	^Ha^ 40.14 ± 0.05	^Ib^ 35.46 ± 0.08	^Ic^ 31.69 ± 0.04	^Gd^ 30.50 ± 0.05	^Ha^ 40.14 ± 0.03	^Ha^ 39.61 ± 0.05	^Hb^ 37.60 ± 0.05	^Gc^ 32.28 ± 0.05
T2	^Da^ 61.27 ± 0.06	^Gb^ 45.66 ± 0.02	^Gc^ 37.67 ± 0.07	^Cd^ 45.37 ± 0.05	^Da^ 61.27 ± 0.05	^Db^ 61.11 ± 0.03	^Ec^ 50.67 ± 0.06	^Cd^ 48.61 ± 0.04
T3	^Ca^ 62.91 ± 0.05	^Cb^ 60.04 ± 0.03	^Dc^ 50.77 ± 0.04	^Bd^ 45.86 ± 0.09	^Ca^ 62.91 ± 0.04	^Cb^ 61.56 ± 0.07	^Dc^ 53.24 ± 0.05	^Bd^ 49.36 ± 0.03
T4	^Fa^ 49.89 ± 0.05	^Fb^ 46.24 ± 0.07	^Fc^ 41.85 ± 0.05	^Hd^ 23.91 ± 0.05	^Fa^ 49.89 ± 0.05	^Fb^ 48.15 ± 0.08	^Ic^ 24.02 ± 0.02	^Ic^ 24.03 ± 0.06
T5	^Ea^ 59.73 ± 0.03	^Eb^ 53.84 ± 0.05	^Ec^ 43.79 ± 0.08	^Ed^ 41.86 ± 0.03	^Ea^ 59.73 ± 0.03	^Eb^ 56.80 ± 0.03	^Fc^ 49.86 ± 0.05	^Ed^ 43.70 ± 0.25
T6	^Aa^ 63.65 ± 0.02	^Bb^ 62.68 ± 0.04	^Cc^ 58.32 ± 0.05	^Ad^ 52.89 ± 0.07	^Aa^ 63.65 ± 0.02	^Ab^ 63.08 ± 0.05	^Cc^ 61.98 ± 0.03	^Ad^ 54.27 ± 0.06
T7	^Ga^ 45.81 ± 0.07	^Hb^ 43.03 ± 0.05	^Hc^ 30.17 ± 0.05	^Id^ 21.57 ± 0.09	^Ga^ 45.81 ± 0.05	^Ga^ 45.34 ± 0.07	^Gb^ 39.31 ± 0.07	^Hc^ 24.38 ± 0.03
T8	^Ba^ 63.42 ± 0.03	^Dc^ 59.25 ± 0.02	^Ab^ 62.59 ± 0.05	^Fd^ 40.31 ± 0.02	^Ba^ 63.42 ± 0.02	^Gb^ 63.08 ± 0.05	^Bc^ 62.76 ± 0.06	^Fd^ 41.27 ± 0.03
T9	^Aa^ 63.68 ± 0.06	^Ab^ 63.15 ± 0.05	^Bc^ 61.27 ± 0.05	^Dd^ 42.95 ± 0.08	^Aa^ 63.68 ± 0.05	^Ab^ 63.52 ± 0.03	^Ab^ 63.48 ± 0.05	^Dc^ 46.16 ± 0.07
Control A	^Ja^ 9.02 ± 0.05	^Kb^ 7.87 ± 0.06	^Kc^ 7.32 ± 0.03	^Jd^ 7.14 ± 0.05	^Ja^ 9.02 ± 0.03	^Jb^ 8.04 ± 0.05	^Jc^ 7.61 ± 0.08	^Jd^ 6.94 ± 0.03
Control B	^Ia^ 10.89 ± 0.02	^Jb^ 9.17 ± 0.04	^Jc^ 8.10 ± 0.05	^Jd^ 7.25 ± 0.08	^Ia^ 10.89 ± 0.05	^Ib^ 8.84 ± 0.05	^Jc^ 7.61 ± 0.09	^Jd^ 7.12 ± 0.06

Samples: T1 (50gr oil + 50gr microcrystalline + 2.5gr thyme), T2 (50gr oil + 50gr microcrystalline + 5gr thyme), T3 (50gr oil + 50gr microcrystalline + 7.5gr thyme), T4 (50gr oil + 75gr microcrystalline + 2.5gr thyme), T5 (50gr oil + 75gr microcrystalline + 5gr thyme), T6 (50gr oil + 75gr microcrystalline + 7.5gr thyme), T7 (50gr oil + 100gr microcrystalline + 2.5gr thyme), T8 (50gr oil + 100gr microcrystalline + 5gr thyme), T9 (50gr oil + 100gr microcrystalline + 7.5gr thyme), control A (Flaxseed oil) and control B (flaxseed oil + microcrystalline with the ratio of 50:75).

**Table 4:** Changes in the carotenoid (mg/kg) of flaxseed oil powder during storage.

Sample	Room temperature (25 °C)				Refrigerator temperature (4 °C)			
	Day 1	Day 30	Day 60	Day 90	Day 1	Day 30	Day 60	Day 90
T1	^Ha^ 4.94 ± 0.04	^Gb^ 4.60 ± 0.06	^Fc^ 4.41 ± 0.05	^Gd^ 3.36 ± 0.05	Ha 4.94 ± 0.05	Hab 4.82 ± 0.03	Eb 4.77 ± 0.05	Hc 3.30 ± 0.07
T2	^Ga^ 5.33 ± 0.05	^Fb^ 4.82 ± 0.08	^Eb^ 4.74 ± 0.05	^Eb^ 4.70 ± 0.06	Ga 5.33 ± 0.04	Gb 5.12 ± 0.05	Fd 4.28 ± 0.05	Fc 4.50 ± 0.03
T3	^Da^ 7.71 ± 0.03	^Db^ 6.53 ± 0.05	^Dc^ 5.26 ± 0.04	^Dc^ 5.16 ± 0.09	Da 7.71 ± 0.05	Da 7.61 ± 0.06	Cb 6.83 ± 0.06	Cc 5.99 ± 0.05
T4	^Ca^ 8.78 ± 0.04	^Cb^ 7.74 ± 0.04	^Cc^ 6.12 ± 0.07	^Dd^ 5.03 ± 0.06	Ca 8.78 ± 0.06	Cb 8.31 ± 0.03	Bc 7.47 ± 0.05	Ed 4.92 ± 0.05
T5	^Ba^ 9.25 ± 0.02	^Ab^ 8.64 ± 0.02	^Ac^ 7.48 ± 0.06	^Bd^ 6.34 ± 0.25	Ba 9.25 ± 0.04	Bb 9.06 ± 0.06	Bc 7.39 ± 0.02	Bd 6.14 ± 0.05
T6	^Aa^ 9.92 ± 0.05	^Bb^ 8.08 ± 0.08	^Ac^ 7.58 ± 0.02	^Ac^ 7.53 ± 0.05	Aa 9.92 ± 0.04	Aa 9.89 ± 0.05	Ab 7.62 ± 0.04	Ac 7.43 ± 0.05
T7	^Ia^ 4.62 ± 0.06	^Ga^ 4.61 ± 0.04	^Fb^ 4.32 ± 0.04	^Gc^ 3.34 ± 0.06	Ia 4.62 ± 0.05	Ja 4.42 ± 0.02	Gb 4.12 ± 0.05	Hc 3.18 ± 0.05
T8	^Fa^ 5.88 ± 0.03	^Eb^ 5.19 ± 0.06	^Ec^ 4.66 ± 0.05	^Fd^ 4.40 ± 0.05	Fa 5.88 ± 0.03	Fa 5.87 ± 0.05	Eb 4.65 ± 0.03	Gc 4.32 ± 0.05
T9	^Ea^ 7.27 ± 0.03	^Db^ 6.67 ± 0.05	^Bc^ 6.48 ± 0.05	^Cd^ 5.43 ± 0.07	Fa 7.27 ± 0.05	Eb 6.46 ± 0.02	Dc 5.92 ± 0.08	Dd 5.11 ± 0.04
Control A	^Ja^ 4.46 ± 0.05	^Hb^ 3.86 ± 0.05	^Hc^ 3.27 ± 0.06	^Hd^ 2.88 ± 0.45	Ja 4.46 ± 0.05	Kb 3.43 ± 0.07	Ic 2.98 ± 0.05	Id 2.59 ± 0.04
Control B	^Fa^ 5.91 ± 0.05	^Fb^ 4.94 ± 0.04	^Gc^ 3.92 ± 0.04	^Hd^ 2.76 ± 0.05	Fa 5.91 ± 0.05	Ib 4.64 ± 0.05	Hc 3.66 ± 0.04	Id 2.57 ± 0.05

Samples: T1 (50gr oil + 50gr microcrystalline + 2.5gr thyme), T2 (50gr oil + 50gr microcrystalline + 5gr thyme), T3 (50gr oil + 50gr microcrystalline + 7.5gr thyme), T4 (50gr oil + 75gr microcrystalline + 2.5gr thyme), T5 (50gr oil + 75gr microcrystalline + 5gr thyme), T6 (50gr oil + 75gr microcrystalline + 7.5gr thyme), T7 (50gr oil + 100gr microcrystalline + 2.5gr thyme), T8 (50gr oil + 100gr microcrystalline + 5gr thyme), T9 (50gr oil + 100gr microcrystalline + 7.5gr thyme), control A (Flaxseed oil) and control B (flaxseed oil + microcrystalline with the ratio of 50:75).

The pigments of samples increased with the incorporation of thyme powder. The incorporation of 15% thyme powder to the oil fraction of the powdered oil increased the chlorophyll and carotenoid contents by 7 and 2.2 times, respectively. It has been reported that chlorophyll and carotenoid contents of extra virgin olive oil increased, followed by flavoring with thyme [31].

### 3.5 Fatty acid composition

The fatty acid composition of our daily diet has high importance as they provide our need for essential fatty acids. They are two groups of essential fatty acids, namely ω-3 and ω-6. There should be a suitable balance in the daily intake of essential fatty acids, and the preferable ω-6: ω-3 ratio is recently reported to be 1:1 [32]. Unfortunately ω-6 is so high in our daily diet, and there is a necessity to increase daily intake of ω_3_ fatty acids such as linolenic acid. As flaxseed is a rich source of linolenic acid, producing edible items rich in flaxseed oil with reasonable stability and shelf life can be a valuable product with a good position in the market. Generally, storage in the refrigerator (4 °C) and also using thyme in the production of flaxseed oil could prevent oxidation and further decrease polyunsaturated fatty acids such as linoleic and linolenic acids (Table 5). Linolenic acid in the flaxseed oil was decreased at a level of 10% during storage which was the highest among samples. Results show that converting flaxseed oil to powder could preserve essential fatty acids and can be a suitable procedure for introducing flaxseed oil to the market. It was reported that any method of capsulation could protect the oil from oxidation during storage [33]. The incorporation of thyme powder also was effective in the reduction of fatty acid oxidation, which was also revealed by PV results as well.

**Table 5:** Changes in the fatty acids (%) of flaxseed oil powder during storage.

Fatty acid	Palmitic acid C16-0			Stearic acid C18-0			Oleic acid C18-1			Linoleic acid C18-2			Linolenic acid C18-3		
Day	1	90		1	90		1	90		1	90		1	90	
Samples		4 °C	25 °C		4 °C	25 °C		4 °C	25 °C		4 °C	25 °C		4 °C	25 °C
T1	7.5	8.1	8.6	5.4	5.9	6.5	23.2	24.8	25.7	15.1	14.3	13.8	47.0	44.7	44.2
T2	8.3	8.5	9.1	4.6	5.5	6.1	23.7	24.6	26.9	14.5	14.0	12.8	46.8	45.6	44.9
T3	7.2	8.6	9.4	5.3	5.8	6.5	24.6	25.5	26.3	15.2	14.7	13.3	46.5	44.3	43.2
T4	7.7	8.5	8.8	4.8	5.2	5.7	24.1	24.8	25.9	14.7	14.3	13.1	47.2	45.4	44.6
T5	7.8	8.3	8.9	5.1	5.8	6.05	23.3	24.2	24.6	14.9	14.1	13.4	47.5	44.8	45.3
T6	7.2	7.6	7.9	4.5	5.4	5.8	25.4	26.6	27.04	15.8	14.6	13.5	46.7	43.6	44.2
T7	7.1	7.6	8.08	4.6	4.9	5.2	24.4	25.6	26.4	14.8	14.3	14.0	46.2	45.9	44.8
T8	8.1	8.5	9.2	4.4	5.2	5.4	23.7	24.8	25.7	15.1	14.5	13.8	46.8	44.3	45.5
T9	8.2	9.4	9.7	4.2	4.6	4.9	23.1	24.05	24.2	15.2	14.6	14.3	47.9	46.1	45.4
Control A	7.9	9.8	8.9	5.4	6.1	6.01	23.1	25.04	25.3	14.6	13.08	14.4	47.7	43.5	42.9
Control B	7.5	9.5	9.9	5.2	6.7	7.3	24.5	26.2	25.8	14.7	13.2	12.5	47.2	43.1	42.5

Samples: T1 (50gr oil + 50gr microcrystalline + 2.5gr thyme), T2 (50gr oil + 50gr microcrystalline + 5gr thyme), T3 (50gr oil + 50gr microcrystalline + 7.5gr thyme), T4 (50gr oil + 75gr microcrystalline + 2.5gr thyme), T5 (50gr oil + 75gr microcrystalline + 5gr thyme), T6 (50gr oil + 75gr microcrystalline + 7.5gr thyme), T7 (50gr oil + 100gr microcrystalline + 2.5gr thyme), T8 (50gr oil + 100gr microcrystalline + 5gr thyme), T9 (50gr oil + 100gr microcrystalline + 7.5gr thyme), control A (Flaxseed oil) and control B (flaxseed oil + microcrystalline with the ratio of 50:75).

As there should not be an effective and strong chemical bonds among flaxseed oil, thyme powder and microcrystalline cellulose, this product consumption and finally digestion in the body can happen as other foods with oil in their formulation.

## 4 Conclusion

Production of flaxseed oil powder, as a rich source of ω-3 essential fatty acids, was successfully achieved by mixing with microcrystalline cellulose. Thyme powder, as a natural source of antioxidants, increased the bioactive compounds content, and was very useful in delaying oil oxidation and also reduction of essential fatty acids. Results of this research showed that transforming flaxseed oil to the powder form, fortified with thyme, was able to control Deteriorating reactions and, thus, enhance oxidative stability. The produced flaxseed oil powder has the potential to be used directly on salads or for the supplementation of food products.

## Author contribution statement

Mahsa Farhoudpour: Methodology, Investigation, Writing - Original Draft.

Mehdi Gharekhani: Methodology, Investigation, Validation, Writing - Review & Editing, Project administration.

Sodeif Azadmard-damirchi: Conceptualization, Methodology, Investigation, Validation, Writing - Review & Editing, Project administration.

Narmela Asefi: Validation, Writing - Review & Editing, Project administration.

## Data availability statement

Data included in article/supplementary material/referenced in article.

## Declaration of competing interest

The authors declare that they have no known competing financial interests or personal relationships that could have appeared to influence the work reported in this paper
